# Supplementary material for: In Vitro Modelling of Oral Microbial Invasion in the Human Colon
Source: Microbiol Spectr. 2023 Mar 27;11(2):e04344-22. doi: 10.1128/spectrum.04344-22 (PMC10100946; doi:10.1128/spectrum.04344-22)
Supplement: Supplemental file 4 — Fig. S1 to S5. Download spectrum.04344-22-s0004.pdf, PDF file, 0.7 MB [file spectrum.04344-22-s0004.pdf]

Supplemental data

In vitro modelling of oral microbial invasion in the human colon.

Authors: Lucie Etienne-Mesmin[1]*, Victoria Meslier[2]*, Ophélie Uriot[1], Elora Fournier[1], Charlotte Deschamps[1], Sylvain Denis[1], Aymeric David[2], Sarah Jegou[2], Christian Morabito[2], Benoit Quinquis[2], Florence Thirion[2], Florian Plaza Oñate[2], Emmanuelle Le Chatelier[2], S. Dusko Ehrlich[2], Stéphanie Blanquet-Diot[1§], Mathieu Almeida[2§].

[1] UMR 454 UCA-INRAE Microbiologie Environnement DIgestif et Santé (MEDIS), Université Clermont Auvergne, 63000 Clermont-Ferrand, France.

[2] Université Paris-Saclay, INRAE, MetaGenoPolis (MGP), 78350 Jouy-en-Josas, France.

* Equal contribution

[§] **Corresponding Authors:** mathieu.almeida@inrae.fr ; stephanie.blanquet@uca.fr

FIG S1. Gut microbiota fermentation activities in M-ARCOL

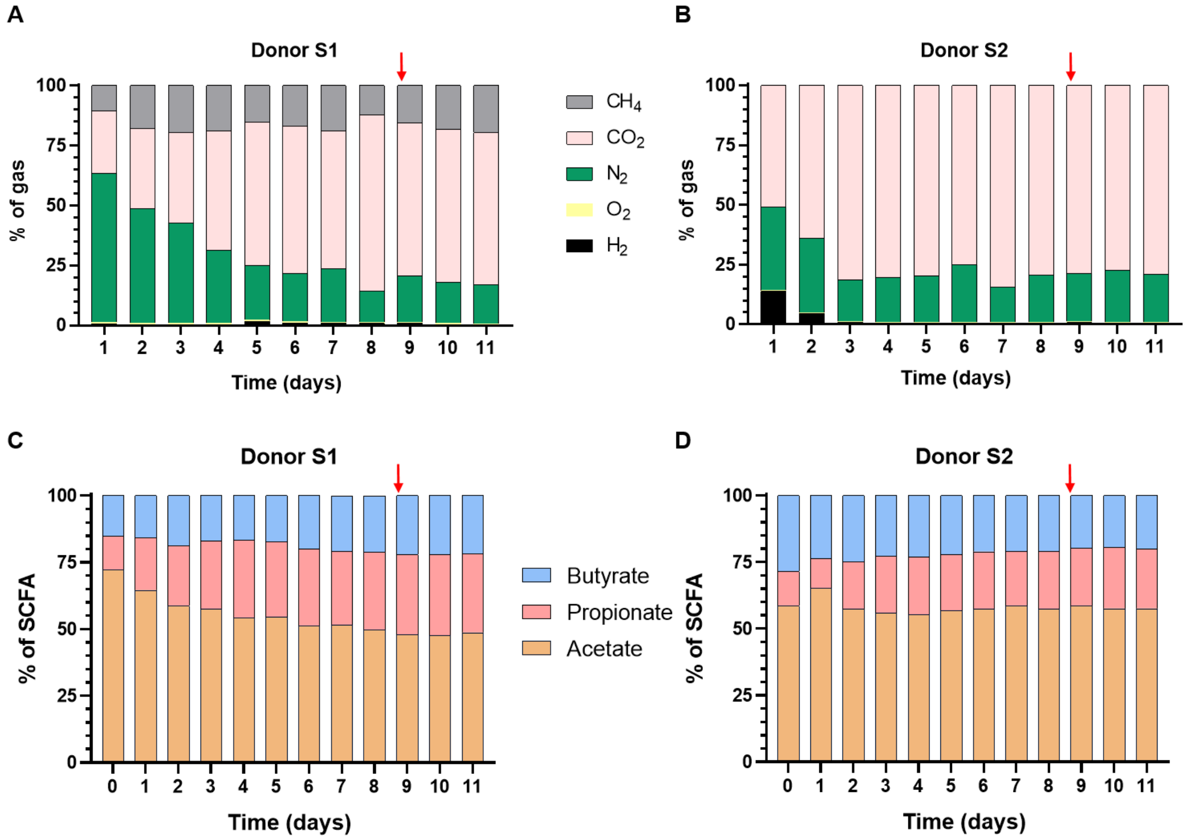

A-B. Gas proportions (O_2, CH_4, N_2, CO_2 and H_2) were analyzed daily in the atmospheric phase of M-ARCOL by gas chromatography throughout the total duration of the fermentation for donor S1 (**A**) and S2 (**B**) and results were expressed as relative percentages.

C-D. Main SCFAs (acetate, propionate and butyrate) were analyzed daily in the luminal compartment of M-ARCOL by high performance liquid chromatography throughout the total duration of the fermentation for donor S1 (**C**) and S2 (**D**) and results were expressed as relative percentages.

The red arrows indicate saliva injection into the bioreactors.

FIG S2. Overview of the mapping rate on the IGC2 10.4M gut and 8.4M oral gene catalogs

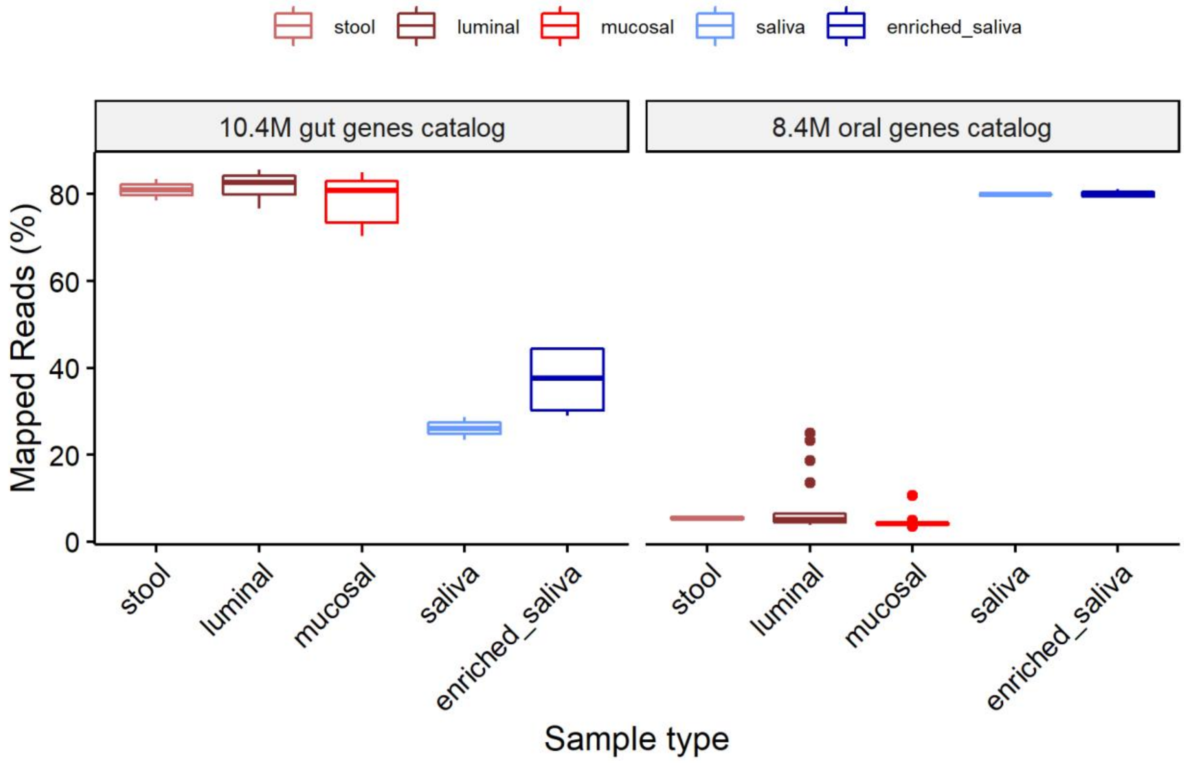

The metagenomic shotgun reads were aligned onto a 10.4 gut microbial gene catalog and a 8.4 oral microbial gene catalog to assess the percent of oral or gut microbial genes detected in the collected microbial samples.

FIG S3. Clustering based on Bray-Curtis dissimilarity distances obtained from the MSP species abundance table

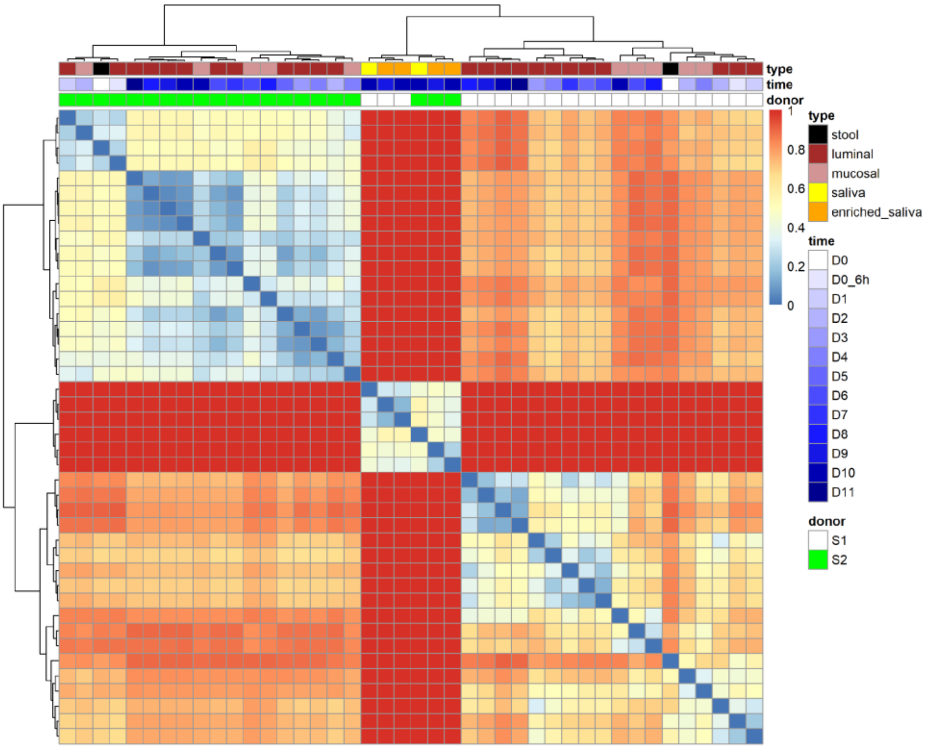

Differences between samples were evaluated by computing the Bray-Curtis dissimilarities based on microbial species abundances for all donors, biosample type (fecal, saliva, bioreactor luminal or mucosal compartment) and time points (from D0 to D11).

FIG S4. Family rank normalized composition

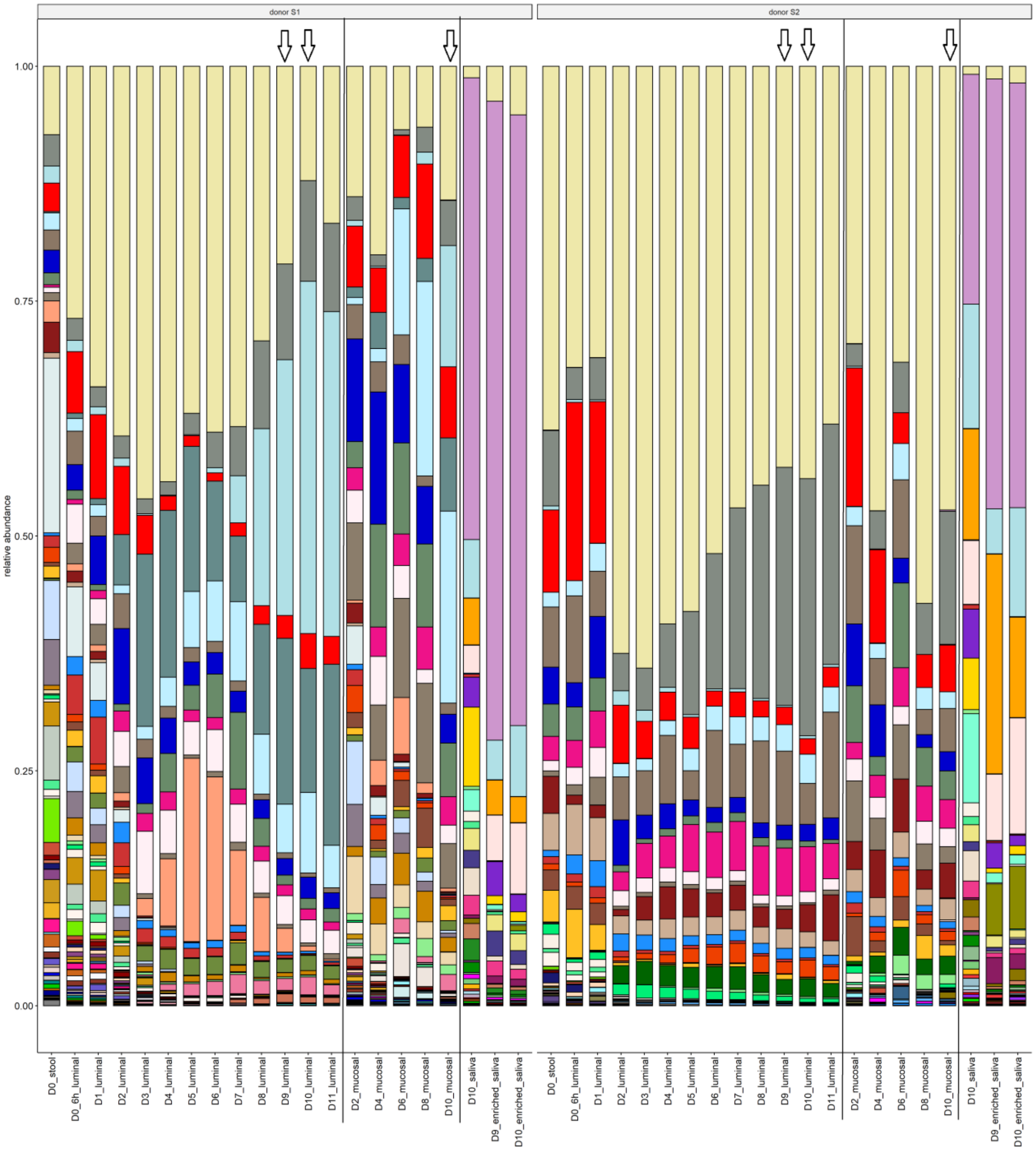

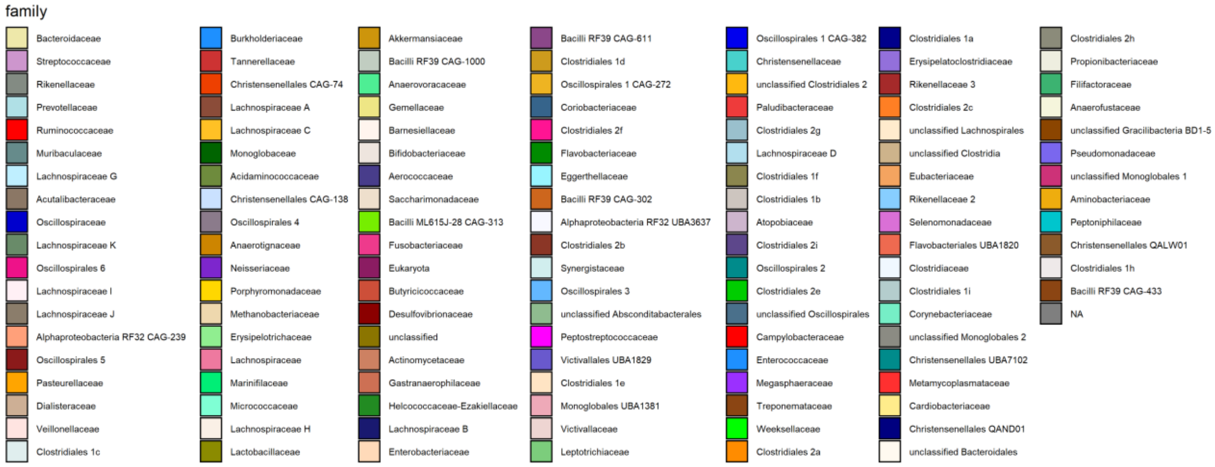

MSP species abundance was normalized per sample by dividing its abundance by the sum of the MSP species abundances detected in the sample. Family rank composition was calculated as the sum of the normalized abundances of the corresponding MSP species.

FIG S5. Oral-to-gut invasion using relative abundance of each ecological niche

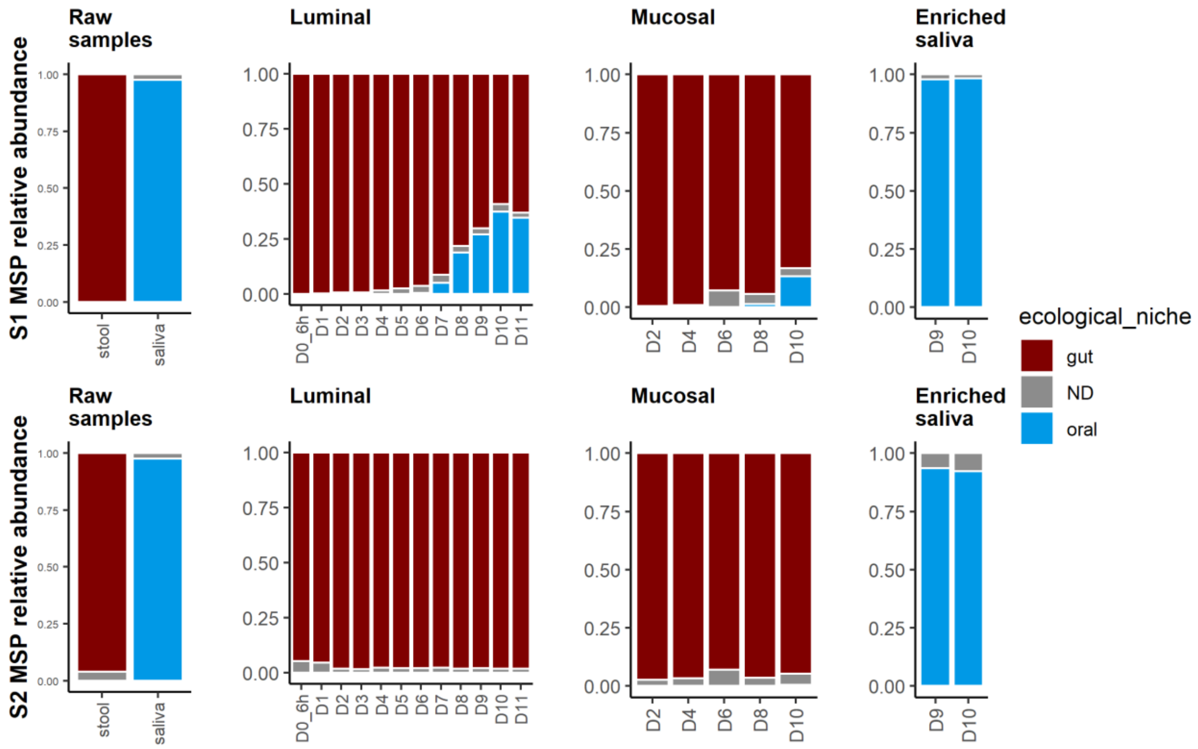

The relative abundance of each ecological niche was determined by adding up MSP species relative abundances based on their respective ecological niche (brown for gut; grey for Not Determined; blue for oral).
